# A mixed-method approach to generate and deliver rapid-cycle evaluation feedback: lessons learned from a multicenter implementation trial in pediatric surgery

**DOI:** 10.1186/s43058-023-00463-x

**Published:** 2023-07-18

**Authors:** Salva N. Balbale, Willemijn L. A. Schäfer, Teaniese L. Davis, Sarah C. Blake, Sharron Close, Gwyneth A. Sullivan, Audra J. Reiter, Andrew J. Hu, Charesa J. Smith, Maxwell J. Wilberding, Julie K. Johnson, Jane L. Holl, Mehul V. Raval

**Affiliations:** 1 Division of Gastroenterology and Hepatology, Department of Medicine, Northwestern University Feinberg School of Medicine, Chicago, IL 60611 USA; 2 Center for Health Services and Outcomes Research, Institute of Public Health and Medicine, Northwestern University Feinberg School of Medicine, Chicago, IL USA; 3 Department of Surgery, Northwestern Quality Improvement, Research, & Education in Surgery (NQUIRES), Northwestern University Feinberg School of Medicine, Chicago, IL USA; 4 Center of Innovation for Complex Chronic Healthcare, Health Services Research & Development, Jr. VA Hospital, Edward Hines, Hines, IL 60141 USA; 5 Center for Research and Evaluation, Kaiser Permanente Georgia, Atlanta, GA USA; 6 Department of Health Policy and Management, Rollins School of Public Health, Emory University, Atlanta, GA USA; 7 Department of Pediatric Advanced Practice Nursing, Nell Hodgson Woodruff School of Nursing, Emory University, Atlanta, GA USA; 8 Division of Pediatric Surgery, Department of Surgery, Northwestern University Feinberg School of Medicine, Ann & Robert H. Lurie Children’s Hospital of Chicago, Chicago, IL USA; 9 Division of Pediatric Surgery, Department of Surgery, Rush University Medical Center, Chicago, IL USA; 10 Department of Neurology, Biological Sciences Division and Center for Healthcare Delivery Science and Innovation, University of Chicago, Chicago, IL USA

**Keywords:** Rapid evaluation feedback, Pediatric surgery, Implementation science, Mixed methods

## Abstract

**Background:**

Rapid-cycle feedback loops provide timely information and actionable feedback to healthcare organizations to accelerate implementation of interventions. We aimed to (1) describe a mixed-method approach for generating and delivering rapid-cycle feedback and (2) explore key lessons learned while implementing an enhanced recovery protocol (ERP) across 18 pediatric surgery centers.

**Methods:**

All centers are members of the Pediatric Surgery Research Collaborative (PedSRC, www.pedsrc.org), participating in the ENhanced Recovery In CHildren Undergoing Surgery (ENRICH-US) trial. To assess implementation efforts, we conducted a mixed-method sequential explanatory study, administering surveys and follow-up interviews with each center’s implementation team 6 and 12 months following implementation. Along with detailed notetaking and iterative discussion within our team, we used these data to generate and deliver a center-specific implementation report card to each center. Report cards used a traffic light approach to quickly visualize implementation status (green = excellent; yellow = needs improvement; red = needs significant improvement) and summarized strengths and opportunities at each timepoint.

**Results:**

We identified several benefits, challenges, and practical considerations for assessing implementation and using rapid-cycle feedback among pediatric surgery centers. Regarding potential benefits, this approach enabled us to quickly understand variation in implementation and corresponding needs across centers. It allowed us to efficiently provide actionable feedback to centers about implementation. Engaging consistently with center-specific implementation teams also helped facilitate partnerships between centers and the research team. Regarding potential challenges, research teams must still allocate substantial resources to provide feedback rapidly. Additionally, discussions and consensus are needed across team members about the content of center-specific feedback. Practical considerations include carefully balancing timeliness and comprehensiveness when delivering rapid-cycle feedback. In pediatric surgery, moreover, it is essential to actively engage all key stakeholders (including physicians, nurses, patients, caregivers, etc.) and adopt an iterative, reflexive approach in providing feedback.

**Conclusion:**

From a methodological perspective, we identified three key lessons: (1) using a rapid, mixed method evaluation approach is feasible in pediatric surgery and (2) can be beneficial, particularly in quickly understanding variation in implementation across centers; however, (3) there is a need to address several methodological challenges and considerations, particularly in balancing the timeliness and comprehensiveness of feedback.

**Trial registration:**

NIH National Library of Medicine Clinical Trials. ClinicalTrials.gov Identifier: NCT04060303. Registered August 7, 2019, https://clinicaltrials.gov/ct2/show/NCT04060303

Contributions to the literature

- Methodological innovations such as rapid-cycle evaluation feedback can potentially help to accelerate implementation in pediatric surgery, where the translation of research evidence into practice has been slow.
- From our experiences in conducting a multicenter trial in pediatric surgery, we learned that using rapid-cycle evaluation feedback is feasible and can be beneficial, particularly in quickly understanding variation in implementation across centers and delivering actionable feedback.
- As a complement to traditional evaluations of implementation, rapid-cycle feedback loops may be an innovative strategy to provide constructive and timely information to enhance implementation while actively engaging members of center-wide implementation teams.

## Background

The implementation of evidence-based interventions, particularly as part of multicenter initiatives, is challenging in healthcare. Although many interventions have similar goals, such as streamlining care delivery processes, improving patient outcomes, and reducing costs, they are, in many cases, as complex as the healthcare delivery problem being addressed. The contexts in which they are implemented can also be complicated, involving multiple interactions within and across stakeholder groups, clinical settings, and care delivery systems [1]. For such multicenter initiatives, providing feedback to individual centers as part of a broader evaluation of implementation is key to facilitate continuous improvement and, ultimately, to maximize intervention effectiveness [2–4]. To achieve robust and sustainable implementation, intervention teams at individual centers also need to be reflexive, or reflective of their own perceptions and actions, and actively engaged throughout implementation and corresponding evaluations [5].

Evidence suggests that rapid-cycle feedback, in particular, can be a valuable strategy in delivering center-level evaluations while fostering both reflexivity and active engagement [6–8]. From their use in healthcare and other fields including education [9–11], we know that rapid-cycle feedback provides timely information as part of efforts to evaluate the uptake of interventions during an implementation period [12, 13]. Cornerstones of rapid-cycle feedback are that (1) the timing and frequency of feedback is often as important as its accuracy and that (2) sharing findings from these assessments in systematic and ongoing “loops” can encourage intervention teams to make iterative changes at their center to enhance implementation early on [13, 14]. From a small but growing literature, we know that delivering rapid-cycle feedback, as part of an implementation evaluation, can be instrumental in observing change over time, overcoming common limitations of one-time observations in traditional research [15, 16]. It can also be helpful in improving the efficiency of implementation and better understanding the unique contextual factors influencing implementation teams through their active involvement [14].

Rapid-cycle feedback may be especially beneficial when applied in clinical specialties such as pediatric surgery, where efforts to translate research evidence into practice have been slower compared to the adult setting [17, 18]. There are several possible reasons underlying this delay. First, children represent a complex and heterogenous population, often with age-specific needs in surgery, which can make implementation more challenging [19, 20]. Other overarching barriers include clinician resistance to change long-standing surgical practices and poor perceived quality of evidence supporting new interventions for children undergoing surgery [15, 21, 22]. From our prior work, which examined early adoption of a bundled, multicomponent enhanced recovery protocol (ERP) to streamline postoperative recovery in hospital-based pediatric surgical programs (referred to as “pediatric surgery centers” hereafter), we also learned that these barriers may contribute to a wide variation in the extent of implementation [23, 24]. To optimize the uptake of interventions, coordinated efforts are needed that acknowledge the unique challenges within pediatric surgery centers while also promoting awareness, enthusiasm, and support for implementation teams to drive change [25–28]. Ongoing feedback that is tailored for pediatric surgery and delivered in rapid cycles may be a helpful facilitator to this end.

There have been some studies of rapid implementation, primarily of telehealth care services, in pediatric surgery and the pediatric care setting more broadly [29, 30], There was also a recent investigation of a digital platform to provide timely assessments on pediatric surgical trainee performance [31]. To date, however, there remains a gap in knowledge about rapid-cycle feedback to support implementation of large, multicenter interventions in pediatric surgery. Drawing on our experiences in implementing a bundled ERP across 18 pediatric surgery centers in the USA, the purpose of this methodological article is to describe our efforts in providing centers with rapid-cycle feedback. All centers are members of the Pediatric Surgery Research Collaborative (PedSRC, www.pedsrc.org), participating in the ENhanced Recovery In CHildren Undergoing Surgery (ENRICH-US) (R01 HD0993440) trial; centers are located within freestanding children’s hospitals and nested within adult hospitals across the USA. We first provide background on the ENRICH-US trial and then share our mixed-method approach for generating and delivering rapid-cycle evaluation feedback to the participating centers based on their implementation efforts. Finally, we reflect on our data collection, preparation, and analytic approach to explore key lessons learned about delivering such feedback in this setting.

## Methods

### The ENRICH-US trial

ENRICH-US, a prospective, multicenter implementation trial, seeks to evaluate the effect of an evidence-based ERP adapted specifically for pediatric surgical patients undergoing elective gastrointestinal surgery. The ERP consists of 21 individual components (presented in Table 1), many of which are similar to adult ERP components. These components include perioperative counseling and education, maintaining euvolemia through limited perioperative fasting, limited intra-operative fluid resuscitation, early enteral intake and mobilization, and limiting opioid use [24, 32]. The components span the pre-admission, pre-operative, intra-operative, and post-operative stages of surgery. While each ERP component, independently, is relatively simple, their combination requires contextually adapted, coordinated efforts across multiple clinical care teams at each stage of surgery [33].

**Table 1:** List of the 21 ERP elements

Preoperative	Intraoperative	Postoperative
1. Patient and family education and engagement 2. Patient Advocate Liaison (PAL) engagement 3. Provider education 4. Optimize medical comorbidities 5. Avoid prolonged fasting 6. Administer non-opioid analgesia	7. Venous thromboembolism prophylaxis 8. Pre-incision antibiotic prophylaxis 9. Standardized anesthetic protocol 10. Surgical procedure (i.e., minimally invasive techniques) 11. Prevention of nausea/vomiting 12. Avoiding nasogastric tubes 13.0Standardized hypothermia prevention	14. No intraperitoneal/perianastomotic drains 15. Goal directed/near-zero fluid therapy 16. Avoiding or early removal of urinary drains 17. Prevention of ileus through gut stimulation 18. Opioid sparing pain regimen 19. Early oral nutrition 20. Early mobilization 21. Audit protocol compliance/outcomes

The ENRICH-US trial is characterized as a type II hybrid stepped-wedge, cluster-randomized study design with three clusters of six pediatric surgical centers. Data are primarily gathered from existing data sources including electronic health records during three phases: baseline, implementation (12 months), and sustainment. The main outcomes of interest are length of hospital stay and, for the implementation evaluation, adoption, fidelity, and sustainability of the ERP. To support team engagement, site principal investigators (PIs) and research coordinators at each center created a *center implementation team*, including multi-professional representatives from pediatric surgery, anesthesia, nursing, child life, patient advocacy, and hospital-level quality improvement (QI). Center implementation teams participated in monthly ERP learning collaborative sessions during the 12-month implementation period, facilitated by the ENRICH-US coordinating center (referred to as “the ENRICH-US team” hereafter), which offered practical guidance and benchmarking of predetermined implementation milestones and center-specific quarterly data reports tracking patient-level ERP compliance as well as benchmarking against peer performance.

For the purposes of this article, we focus only on the methodological aspects of the ENRICH-US trial that were relevant to our rapid-cycle evaluation feedback process. This study was approved by Northwestern University’s Institutional Review Board. Further details on the ENRICH-US trial are published elsewhere [33].

### Rationale and operationalization of rapid-cycle feedback

The ENRICH-US team, composed of the multiple principal investigators (MPIs), co-Is, research staff, and pediatric surgery fellows with diverse expertise in surgical care delivery; quantitative, qualitative, and mixed methods; implementation science; health services and outcomes research; and pediatrics, held scheduled meetings to discuss emerging and ongoing topics related to the implementation of the ERP at the 18 participating pediatric surgery centers. During these meetings, we established a need to provide each center with a brief, actionable update on their progress with implementation at the halfway point (6 months) and at the end of their 12-month implementation period. Review of prior recommendations on rapid-cycle evaluation feedback [15, 34] and feedback from the ENRICH-US team led to the creation of a 1-page implementation report card, delivered electronically to each center, as the platform for delivering the feedback. Each center’s implementation team was the target audience for the report card with the goal of providing the team with a clear understanding of their implementation status based on our assessment as well as their key strengths and areas in need of implementation improvement.

### Study design and data collection

To assess implementation efforts and complete the report cards, we conducted a mixed-method sequential explanatory evaluation [35], consisting of quantitative surveys and in-depth, qualitative interviews with each center’s implementation team. A summary of our mixed method approach is presented in Table 2. Data collection began in September 2021 and is currently underway for the trial’s third and final cluster. First, a 17-item cross-sectional survey was administered, electronically, to all centers at the 6-month timepoint of their implementation period. The site PI or other representative of the individual center’s implementation team was asked to complete the survey. The survey questions were developed and refined by the ENRICH-US team and focused on details about implementation of each individual component of the ERP (e.g., recruiting a patient/family liaison for the implementation team, creation of tools and materials to educate patients, and families about the ERP; frequency of center implementation team meetings and participation of team members; and challenges and resistance of clinicians to implementation of the ERP components). The survey was specifically designed to be low-cost and low-burden for survey respondents yet able to detect major implementation accomplishments and problems at the centers [8, 12].

**Table 2:** Summary of mixed method data collection

Research method	Timing and sample	Purpose
Site surveys (quantitative)	Brief survey (15 multiple choice questions) administered at 6 months and then again at 12 months following the start of center’s implementation period Sample: Site PIs or other representatives of each center’s implementation team	Gain understanding of the center’s ongoing progress and the extent to which centers have implemented the intervention Gain a baseline understanding of key strengths and weaknesses of the center’s implementation progress
In-depth interviews or focus groups	In-depth, semi-structured interviews or focus groups conducted as a follow-up to each center’s site survey results at 6 months and then again at 12 months following the start of center’s implementation period Sample: Members of each center’s implementation team (i.e., a pediatric surgeon/site PI; other clinical team members such as anesthesiologists and nurses; study coordinator	Gain understanding of the center’s implementation processes, challenges, facilitators, and opportunities for improvement from the perspective of center’s own implementation team

Second, a 1-h semi-structured interview was conducted with site PIs (predominantly pediatric surgeons) and any additional available implementation team members (e.g., study coordinators, anesthesiologists, gastroenterologists, nurses, nurse practitioners, physician assistants, and patient advocate liaisons). For each center, the interview was conducted as soon as possible following receipt of their completed survey. The interviews were conducted via video-conferencing to accommodate schedules and time differences. Each interview typically had two interviewers from the ENRICH-US team, including a qualitative researcher and a surgeon to address any clinical issues. Participants provided verbal consent to participate and to be recorded. Key topics were selected for discussion during the interview, including data collection and understanding facilitators and barriers to implementation of the individual ERP components as well as potential strategies or workarounds used by centers to overcome barriers. Interviews conducted at the 6-month timepoint of implementation focused on early implementation experiences, whereas interviews at the 12-month timepoint examined the overall implementation experience and any plans to sustain implementation beyond the center’s implementation period. Prior to conducting the interview, the interviewers reviewed the center’s survey results to inform the interview discussion. As recommended in prior studies, we maintained a focus on collecting data quickly with detailed notetaking [8, 13]. For each interview, both interviewers participated in notetaking and used a targeted approach to gather information on potential underlying factors that shaped the center’s implementation experience and would be relevant in preparing the center’s implementation report card. All interviews were audio-recorded and transcribed verbatim professionally. In most cases, however, implementation report cards were prepared and delivered to the respective center before the transcripts were ready; therefore, interviewers relied on their notetaking, any relevant team discussion, and the audio recording of a center’s interview in preparing a report card.

### Rapid-cycle evaluation feedback strategy

**Fig. 1:**
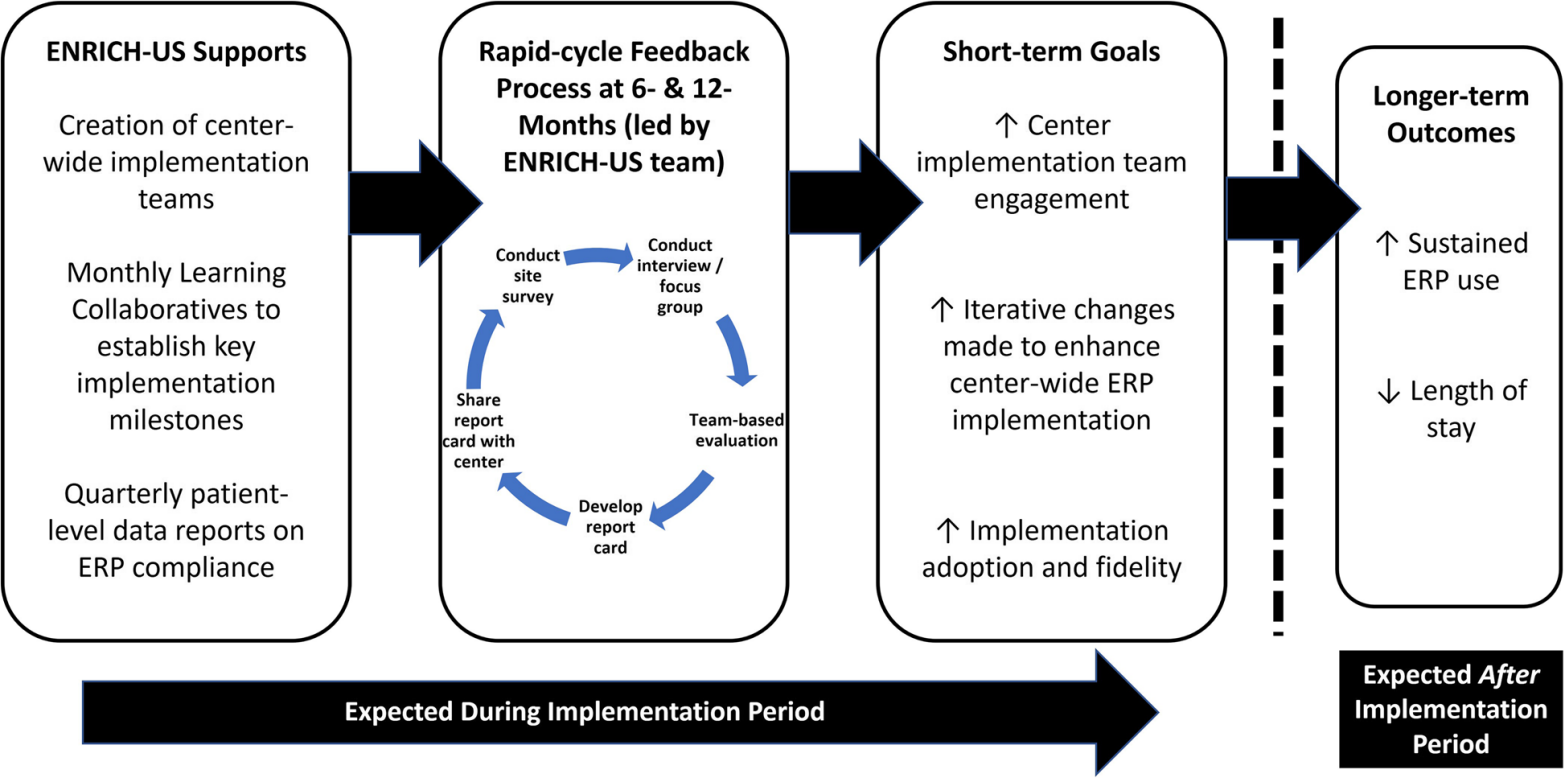
Framework for rapid-cycle evaluation feedback for ENRICH-US (adapted from Zakocs et al.)

**Fig. 2:**
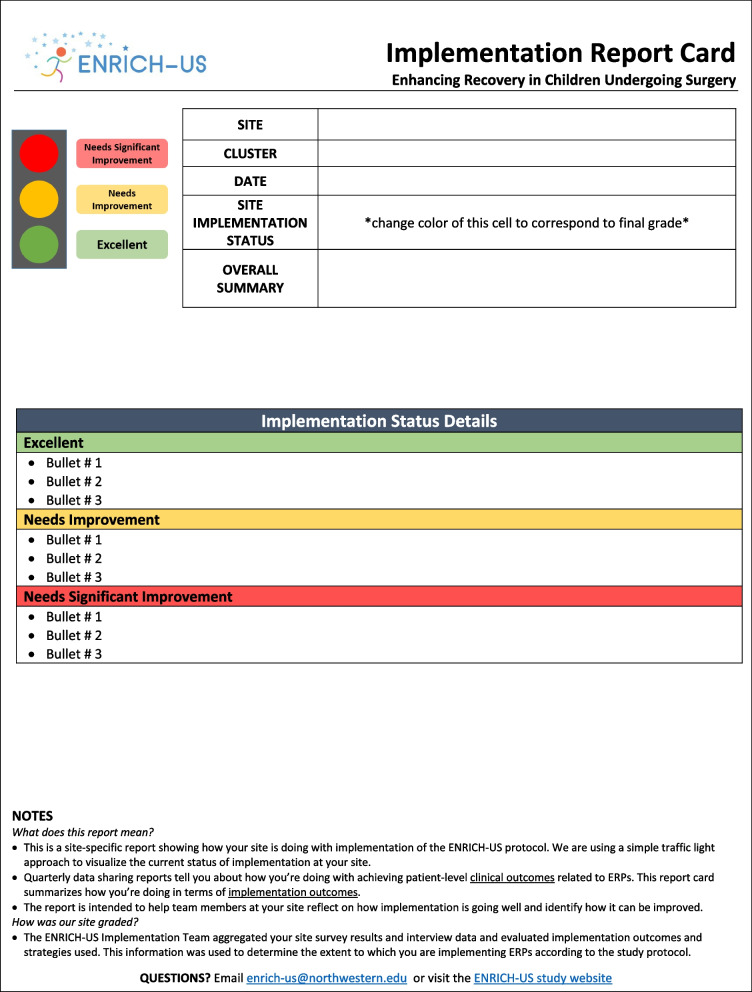
Implementation report card template

**Fig. 3:**
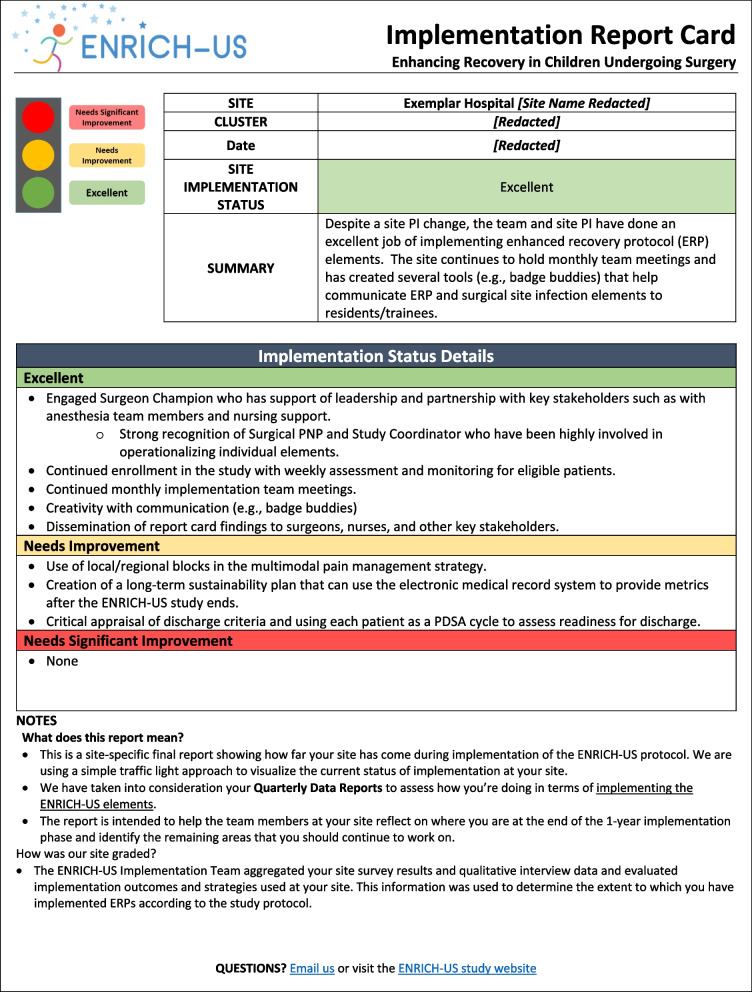
Exemplar implementation report card

We reviewed prior studies describing the steps needed to conduct rapid-cycle evaluations and deliver corresponding feedback [14, 15, 34]. Based on this literature, and accounting for the main goals of the ENRICH-US trial, we adapted a framework previously established by Zakocs et al. for rapid-cycle evaluation feedback (Fig. 1) [8]. Following guidance from prior work in this area [8, 14, 16], we leveraged rapid evaluation techniques and used our detailed notetaking and iterative discussion within the team to provide feedback to the center within 1 week of the interview. For each center, we triangulated the survey and interview data with their most recent quarterly report to complete their report card. The report card was prepared by the ENRICH-US team members who conducted the interview. The final implementation report card template is shown in Fig. 2 and, as an exemplar, a completed report card is included in Fig. 3. As in other studies, report cards used a “traffic light” approach to quickly visualize the center’s overall implementation status at the given timepoint, where green = excellent; yellow = needs improvement; and red = needs significant improvement [36, 37]. Report cards summarized key strengths and opportunities for implementation improvement and were sent electronically to all members of a center implementation team within 10 days of the interview.

## Results

Data collection and creation of the report card feedback occurred at the 6-month and 12-month time points of the 12-month implementation period, which will yield 36 surveys and 36 interviews. An overview of the key steps to generate and deliver the report card feedback is shown in Table 3.

**Table 3:** Five steps for implementing rapid-cycle evaluation feedback using implementation report cards in pediatric surgery (adapted from Zakocs et al.)

Step	Considerations
1. Clarify intent and action plan	• Identify team members at the coordinating center who will be involved in the rapid-cycle feedback process • Ensure that pediatric surgery clinical team members are included in each step • As a team, discuss the purpose and align on the protocol to generate and deliver rapid-cycle feedback • Draft a 1-page implementation report card template and discuss what fields this should include • Align on who the target audience is for report cards (e.g., center implementation teams) and what they should ultimately take away from a completed report card
2. Collect “good enough data”	• List key questions that should be addressed in data collection, keeping the implementation report card fields in mind • Identify low-cost data collection strategies and describe who will do what in a timely manner (Data collected through existing programmatic channels are preferred) • Collect data quickly and with detailed notetaking
3. Engage in team-based evaluation and discussion	• As a team, interpret each center’s data quickly as it is collected (e.g., using team-based discussion and/or targeted review of data and notes) within 1 week of data collection • Engage in a reflective discussion with team about findings that should be highlighted in implementation report card. Center discussion around three questions: • What are we learning about this pediatric surgery center’s efforts to implement the intervention? (What?) • For this center, what are the likely implications of our findings? (So what?) • What actions are required to improve implementation moving forward? (What now?) • Results should be certain enough for center implementation teams to make decisions about adjustments to their implementation efforts
4. Develop implementation report card as a team	• As data for each center is collected/interpreted, draft a center-specific implementation report card that highlights the major findings only (leave out the details) • Ensure the report card is visually appealing (e.g., using color, pictures, and graphics) • Share completed implementation report card with other team members within the coordinating center for internal review before it is final
5. Share report cards directly with respective centers	• Distribute the final version of the implementation report card via email within 10 days after data have been collected • Share report cards directly with each pediatric surgery center’s implementation team, including the site PI

Following discussion and reflection within the ENRICH-US team on our experience applying this methodology, we identified several practical lessons for using rapid-cycle feedback about multi-center implementation of an intervention. The lessons are grouped according to three broad categories, including the (1) potential value/benefits of the approach, (2) potential challenges, and (3) practical considerations for providing rapid-cycle evaluation feedback. Key lessons within each category are described below.

### Category 1: potential value and benefits of rapid-cycle evaluation feedback

*Benefits lesson #1*: The approach enabled *quick understanding of variation in implementation and needs across centers*. The extent to which centers were actively engaged in implementing and adhering to the ERP components was widely variable, anecdotally, from comments made during monthly learning collaborative meetings. However, the mixed method evaluation provided rapid evidence on the varying extent of implementation between centers and was a valuable data source and supplement to each center’s quarterly data report.

*Benefits lesson #2*: The report card *delivered actionable feedback efficiently* to centers. Given delivery of the report cards within 10 days of the interview, centers received actionable feedback on their implementation status. For example, at the 6-month time point, some centers reported challenges in identifying and recruiting eligible patients. In response, we included strategies and resources, from other centers, that were successful for identifying and recruiting patients.

*Benefits lesson #3*: The approach *facilitated partnerships with individual centers*. The structure and timeline of the rapid-cycle process, including data collection and delivery of the report cards, served as opportunities to repeatedly engage the center implementation teams. Although centers participated in the monthly learning collaborative meetings, the individual center interviews and feedback process allowed for a more targeted and tailored discussion with each center about their implementation efforts. Through this process, the ENRICH-US team was able to establish themselves as partners with the centers with shared goals regarding implementation. For example, this was particularly helpful to build stronger relationships between the ENRICH-US teams and pediatric surgeon and anesthesia champions at various centers. In some cases, the rapid-cycle process was beneficial in shifting the conversation from a mindset of “What did the center do wrong?” to “In what tangible ways can the center optimize implementation of the ERP?”.

### Category 2: potential challenges associated with rapid-cycle evaluation feedback

*Challenge lesson #1*: The data collection process *requires substantial resources*. Although we did not conduct traditional data analysis of the survey and interview data, substantial time and resources were required to produce a reliable and meaningful report card, given the substantial amount of data, the need for integration of data, and rapid interpretation. The ENRICH-US team members needed to review a center’s survey data and most recent quarterly data report prior to the interview and, then, integrate the interview data. Occasionally, additional team discussions were needed to iteratively refine the key messages to be included in the report card. Substantial resources were needed to achieve our 10-day goal for delivery of the report card, including personnel, time, and methodological skills. The ENRICH-US team consists of 15 individuals, of whom 12 have training and expertise in qualitative research methods and are available to participate in the interview and report card preparation process. Such a large group is unlikely to be available for all projects. Specific research skills are needed to carry out this type of rapid-cycle feedback process, particularly strong qualitative research skills to conduct valid interviews and to integrate multiple sources of data.

*Challenges lesson #2: Reaching consensus is essential*. The methodological rigor associated with traditional qualitative coding and analysis is, in many cases, not possible in the rapid approach given the goal to ensure timeliness. Yet, reaching consensus among the research team members can be used as a strategy to reduce bias. Rapid-cycle feedback is inherently team-based and maintaining alignment among team members throughout the process was essential. Engaging in team-based discussions about the survey and interview data was imperative to ensure that feedback in the report cards was both accurate and constructive. In our view, achieving broader consensus among team members about the content of center-specific feedback enhanced the credibility and validity and reduce potential bias of the report cards. Much like in traditional qualitative research, however, reaching this consensus is an additional step that takes time and can be challenging when coordinating with team members. To achieve consensus efficiently in this study, the ENRICH-US team members who conducted the interviews and drafted the implementation report cards then shared them with all remaining team members. This would prompt any discussion among the broader team and highlight whether other team members agreed with the key points drafted in the implementation report card or had a differing point-of-view. In cases of such differing points-of-view, the report cards were revised until all team members agreed with the content.

### Category 3: practical considerations when providing rapid-cycle evaluation feedback

*Practical consideration #1*: Balancing *timeliness versus comprehensiveness* of feedback can be challenging. Generating and delivering feedback in a rapid cycle required constantly balancing timeliness with comprehensiveness. Because this was a new methodology for most of the ENRICH-US team, team members needed to shift away from traditional data analytic approaches, requiring coding and theme development, and adoption of the rapid-cycle process. This required setting new expectations for ENRICH-US team members. Centers were informed that the rapid feedback was part of participating in the ENRICH-US trial and pitched as a way facilitate implementation improvements.

*Practical consideration #2*: The approach *actively engages members of each center’s implementation team*. For effective implementation, the implementation team needs to be actively engaged. The process of collecting both survey and interview data and, subsequently, creating and delivering the report cards, can uniquely support this engagement. Although limited to the members of each center’s implementation team, the rapid feedback process served as an opportunity to interact directly with each center. In our experience, pediatric surgeons and anesthesia champions, rather than study coordinators, nurses, and QI professionals, participated primarily in the interviews. We reflect that perhaps encouraging more individuals from these other groups (study coordinators, nurses, QI professionals, etc.) to participate in the interviews may have helped in (1) establishing that center implementation teams are clearly multidisciplinary and (2) ensuring that all team members are actively involved in the feedback process.

*Practical consideration #3*: To be successful, *an iterative and reflexive approach is needed*. As part of our deliberate shift away from traditional research methods and toward the methodology used in rapid-cycle evaluation, we found that adopting an iterative, reflexive approach was essential. In this context, it was important to view our relationship with individual centers as long-term that would evolve over time. With data collection and delivery of report cards at two time points within the 12-month implementation period, we were able to observe changes, over time. For example, following our recommendation and provision of strategies, a center recruited a patient advocate liaison, which helped to improve screening for eligible patients. Adopting an iterative and reflexive approach, particularly in our communications with centers appears to promote a culture of ongoing evaluation and self-awareness, while encouraging centers to make quick improvements, both big and small, in response.

## Discussion

In this study, we leveraged a mixed-method approach [35] and previously established evaluation methods [8, 15] to apply rapid-cycle feedback about implementation of an evidence-based pediatric surgery intervention in a multicenter trial. We learned that using a rapid, mixed method approach is feasible and can be beneficial, particularly in quickly understanding variation in implementation across centers and delivering actionable feedback. We also identified potential methodological challenges and considerations when using rapid-cycle feedback. For example, several resources (e.g., personnel, time) and skills are needed to carry out the process. As a complement to more traditional evaluations of implementation, rapid-cycle feedback loops may be an innovative strategy to provide constructive and timely information to enhance implementation while actively engaging members of center-wide implementation teams.

A key observation was related to the inherent tension between providing timely feedback that was also comprehensive and accurate. This required the researchers to consciously shift our mindset from traditional research analytic methods and toward a more consensus-derived, rapid approach. This tension has been observed in other rapid-cycle evaluation studies conducted outside of pediatric surgery [38]. It is possible, in some ways, that this observation reflects a much larger question permeating through implementation science and health services research about how to balance the ubiquitous need to accelerate the scientific process and translate evidence-based interventions much more quickly into practice sooner while maintaining methodological rigor. Although the answer may not be clear, existing literature suggests that we must increasingly focus on expediting efforts to implement and evaluate healthcare interventions [39–42]. We hypothesize, then, that using our rapid-cycle feedback approach in the ENRICH-US trial, and prioritizing timeliness, may be a modest but important step in advancing implementation science methods. And while our focus was primarily on timeliness of the feedback, it was imperative, as we and others have, to lean in across disciplines and adapt established methods to evaluate and deliver rapid-cycle feedback as rigorously as possible [5, 16].

The application of rapid-cycle feedback, including customized implementation report cards, to our knowledge, is new in pediatric surgery. Clinicians and researchers who aim to implement complex, multicenter interventions in this setting should be aware that these are, by definition, versatile methods designed to support implementation by providing centers with the information they need at the right time [12]. In turn, this may promote the awareness and enthusiasm that is essential for intervention teams to drive change [16]. In addition to these lessons from the ENRICH-US experience, other studies have found that using these approaches can help in better aligning the goals of healthcare interventions with the needs of key stakeholders, including the individuals who deliver the intervention and those who receive it [5, 8, 43, 44]. This is perhaps especially important to consider in pediatric surgery, given that an increasingly multidisciplinary group of surgeons, anesthesiologists, and other clinical staff may be implementing any intervention focused on children, who also represent a heterogeneous group with unique needs [20, 21, 45]. Given the successful application of rapid-cycle evaluation feedback in settings outside of pediatric surgery [8–10], ensuring that future rapid-cycle evaluation efforts and reports, such as our report card, are tailored for the pediatric surgery setting will continue to be important. It will also be important to understand whether these rapid evaluation approaches can help in overcoming the major barriers that have been documented in prior research to implementing evidence-based interventions in pediatric surgery [21, 27].

To expand the use of rapid-cycle feedback in pediatric surgery, healthcare QI methodologies might be leveraged. It is possible that some clinicians in pediatric surgical care are more familiar with QI, which usually involves a system-level project to improve the quality, safety, and value of healthcare, rather than implementation science. Although implementation science, as the study of systematic uptake of evidence-based interventions into practice, is a distinct field compared to QI, both implementation science and QI typically involve both qualitative and/or quantitative research methods and share an overlapping goal to drive or evaluate system-level change in healthcare practice [46–48]. This overlap has been previously recognized in surgery [49]. Rapid-cycle evaluation may naturally fit in the middle of this overlap, sharing a similar philosophy with QI around driving continuous improvement locally, while also being characterized as a methodology within implementation science. Rapid assessment procedures, which represent an emerging methodological area within implementation science, have already been established as a pragmatic approach to produce timely and contextually rich evaluative information about complex interventions implemented into dynamic clinical settings [16, 50, 51]. Adapting rapid-cycle efforts so that they use established QI methodologies to continuously improve care, eventually for broader use, could be a helpful strategy to promote buy-in and engagement of this approach in pediatric surgery. This may be especially suited for surgical interventions, such as ERPs, whose foundational principles are predicated on rapid-feedback and continuous process improvement [52].

Our methodological approach is subject to limitations. First, we conducted two rounds of data collection for each center and provided them with a report card twice over their 12-month implementation period. It is possible that conducting the rapid-cycle process on a more frequent basis, for example, quarterly, as in other studies [14], could better promote iterative improvements to implementation and active engagement. It is also possible that carrying out more rapid cycles would have encouraged engagement from other members of center implementation teams, such as QI professionals and patient advocate liaisons, who generally participated less in the data collection. Inviting a multidisciplinary group to participate consistently in the evaluation process may be beneficial and may increase enthusiasm around implementation locally. As described previously, the rapid-cycle methodology, itself, also introduces some limitations by sacrificing a purely inductive approach and focusing, instead, on quickly generating targeted insights from the collected data [8, 14]. We also note that, although we successfully executed our rapid-cycle strategy, we did not seek feedback from the center’s about perceived value of the report cards. We do not know, what, if any, direct changes or improved outcomes were realized as a result of the report cards. This limitation is corroborated in other rapid evaluation studies [8, 10, 12].

## Conclusion

To our knowledge, this is the first example of rapid-cycle feedback applied to a multicenter implementation trial in pediatric surgery. From a methodological perspective, we learned many lessons: (1) using a rapid, mixed method evaluation approach is feasible in pediatric surgery and (2) can be beneficial, particularly in quickly understanding variation in implementation across centers; however, (3) we also identified methodological challenges and considerations, particularly in balancing the timeliness and comprehensiveness of feedback. To complement more traditional evaluation of implementation, rapid-cycle feedback may be an innovative strategy to provide timely and constructive information to enhance implementation of evidence-based interventions in pediatric surgery.

## Data Availability

Data may be available upon request.

## Abbreviations

ENRICH-US: ENhanced Recovery In CHildren Undergoing Surgery trial
ERP: Enhanced recovery protocol
PedSRC: Pediatric Surgery Research Collaborative
PI: Principal investigator
MPI: Multiple principal investigators
